# THE EFFECT OF OLDER ADULTS’ VOLUNTEERING ON THE SOCIAL SUPPORT

**DOI:** 10.1093/geroni/igz038.625

**Published:** 2019-11-08

**Authors:** Meeryoung Kim

**Affiliations:** Daegu University, Daegu, Korea, Republic of

## Abstract

With increasing longevity, older adults need activities after retirement. Volunteering can be alternate for substituting role after retirement. Social capital is an important resource to start volunteering. Vice-versa, volunteering can increase their social network and social support. This study examined how the factors of volunteering affect emotional, instrumental and esteem support of older adults. This study used the 6th additional wave of the Korean Retirement and Income Study (2016). The sample size was 202 and target population were adults age 65+. Multiple regressions were used for data analysis.. Demographic variables (e.g. gender, age, etc) were controlled. Independent variables included volunteer time, how many places they volunteered at, whether volunteers were professionals providing pro-bono services or not, whether they were self-motivated or asked by others. For dependent variables, social support such as emotional, instrumental and esteem support were used. If volunteers were asked by others, emotional and esteem support were increased. If volunteers were self-motivated, it affected esteem support. If they were volunteering in multiple places, instrumental and esteem support were higher than volunteering in only one place. Length of volunteering time spent affected the instrumental support negatively. There were differences between those who were professionals versus nonprofessional volunteering affecting instrumental support. Nonprofessional volunteering affected instrumental support more than professional volunteering. These findings implied social support motivation, time, and whether the volunteer was a professional or not affected different kinds of social support differently. Findings show the importance of older adults doing volunteering to enhance social support.

